# Injury prevention content in social media posts by Children’s hospitals: a missed opportunity for education and advocacy

**DOI:** 10.1186/s40621-026-00705-1

**Published:** 2026-08-14

**Authors:** Maneesha Agarwal, Jacqueline B. Gluck, Zoe Fischman, Harleigh J. Markowitz, Morgan Cantor, Zoë Teicher, Kristyn Jeffries, Wendy J. Pomerantz

**Affiliations:** 1 Department of Pediatrics, Division of Emergency Medicine, Emory University School of Medicine, Atlanta, GA 30322 USA; 2 College of Arts and Sciences, Department of Biology, Emory University, 201 Dowman Dr, Atlanta, GA 30322 USA; 3 College of Arts and Sciences, Department of Psychology, Emory University, 201 Dowman Drive, Atlanta, GA 30322 USA; 4 Department of Health Behavior, University of North Carolina Gillings School of Global Public Health, 135 Dauer Drive, Chapel Hill, NC 27599 USA; 5 Department of Pediatrics, Section of Hospital Medicine, University of Arkansas for Medical Sciences, Arkansas Children’s Hospital, 1 Children’s Way, Little Rock, AR 72202 USA; 6 College of Arts and Sciences, Department of Neuroscience and Behavioral Biology, Emory University, 201 Dowman Drive, Atlanta, GA 30322 USA; 7 Department of Pediatrics, Section of Hospital Medicine, University of Arkansas for Medical Sciences, Arkansas Children’s Hospital, 1 Children’s Way, Little Rock, AR 72202 USA; 8 Division of Pediatric Emergency Medicine, Department of Pediatrics, Cincinnati Children’s Hospital Medical Center, University of Cincinnati College of Medicine, Cincinnati, OH USA; 9 Division of Pediatric Emergency Medicine, Arthur M Blank Children’s Hospital - Children’s Healthcare of Atlanta, 2220 North Druid Hills Road NE, Atlanta, GA 30329 USA

**Keywords:** Social media, Injury prevention, Facebook, Instagram, Twitter

## Abstract

**Background:**

Preventable injuries are the leading cause of pediatric death. Most healthcare organizations and the general public engage in social media (SoMe) to disseminate and consume health-related information. It is unknown how frequently pediatric hospitals leverage their SoMe platforms to educate on injury prevention (IP) topics. We sought to better characterize SoMe messaging and IP content by children’s hospitals.

**Methods:**

This was a retrospective cross-sectional study of US children’s hospitals’ primary SoMe Facebook, Twitter/X, and Instagram accounts. Included hospitals were associated with Injury Free Coalition for Kids (IFCK), a current or past Centers for Disease Control and Prevention Injury Control and Research Center, a level I pediatric trauma center, or a pediatric surgery fellowship. Accounts established after 1/1/23 or covering adult health topics were excluded. Abstractors reviewed all available posts from 2023; posts were dichotomized into IP vs. non-IP content, with further subcategorization based on injury mechanisms and other topics covered. Descriptive statistics and frequencies with ranges were calculated. Chi-square analyses were used for comparisons between groups.

**Results:**

Of 82 unique hospitals with eligible SoMe accounts, all used Facebook and 69 (84.1%) used all 3 SoMe platforms. Of the 55,339 posts, 3,863 (7.0%) posts covered IP. Among IP posts, the most frequently covered specific topics were mental health/suicide, poisonings, and child passenger safety. Most non-IP posts were focused on general publicity/goodwill; other frequently covered medical categories were cardiac conditions, cancer, and neonatal diseases. Hospital affiliation with a pediatric surgery fellowship (***X***^2^ = 93.79; *p*<.001) and IFCK (***X***^2^ = 4.56; *p*=.03) were associated with more IP content. More affiliations with IP-oriented organizations were also associated with more IP content (***X***^2^ = 119.8; *p*<.001).

**Conclusion:**

Although children’s hospitals have large SoMe followings, IP is rarely discussed. While ties to IP-oriented organizations improve coverage of IP content, this represents a critical missed opportunity to address the leading causes of pediatric deaths.

## Background

The rapid expansion of digital technology has driven a substantial increase in social media (SoMe) use. Over 80% of Americans now use at least one SoMe platform, and this number continues to grow [1–2]. Notably, nearly 60% of Americans report using SoMe to obtain health information [3]. This trend includes parents and other caregivers of children, who frequently rely on SoMe for information related to child health and development [4–6]. Collectively, these trends underscore the potential of SoMe as a powerful channel for communicating critical public health information.

Hospitals and healthcare systems have increasingly recognized this potential by expanding their presence on multiple SoMe platforms [7]. These accounts are used for multiple purposes, including public engagement, promotion of services, generating goodwill, employee recognition, fundraising, and dissemination of health information [8–10]. Despite this growth, it remains unclear how effectively healthcare organizations leverage SoMe to address specific public health priorities such as pediatric injury prevention (IP).

Injuries are the leading cause of death and disability for children in the US [11–12], making pediatric IP a critical target for public education. Given the widespread reach and accessibility of SoMe, children’s hospital-based SoMe accounts represent a unique opportunity to enhance public knowledge and promote behaviors that reduce injury risk.

The objective of this study was to characterize SoMe messaging from leading US children’s hospitals, with specific attention to IP messaging, via descriptive analysis of US pediatric hospitals’ SoMe accounts. We hypothesized that fewer than 20% of all SoMe posts would address pediatric IP. We further hypothesized that hospitals with greater affiliation with IP-oriented organizations would post IP-content more frequently than those with fewer such affiliations.

## Methods

We conducted a retrospective, cross-sectional study of SoMe posts from US children’s hospitals in 2023. Eligible hospitals were identified based on affiliation with organizations or designations reflecting a demonstrated commitment to pediatric IP. These included affiliation with Injury Free Coalition for Kids ^®^ (IFCK), a national, evidence-based, hospital-affiliated nonprofit organization dedicated to pediatric IP; current or past designation as a Centers for Disease Control and Prevention (CDC) Injury Control and Research Center (ICRC), representing institutions engaged in advanced IP research, outreach, and education; verification as a level I pediatric trauma center, which requires dedicated IP personnel and programming; and sponsorship of a pediatric surgery fellowship, which entails substantial research infrastructure and academic engagement. Eligibility was determined based on hospital affiliation as of spring 2024.

For each eligible hospital, we identified official accounts on Facebook (FB), Instagram (IG), and X (formerly known as Twitter, hereafter referred to as X). These platforms were selected because they represented the most widely adopted social media platforms among eligible children’s hospitals during the study period. TikTok and YouTube were not included because relatively few hospitals maintained eligible TikTok accounts as of January 1, 2023, and both platforms rely primarily on video-based content that differs substantially from the content formats primarily used on FB, IG, and X in 2023.

Accounts were excluded if they were established after January 1, 2023, or if their content was not exclusively pediatric-focused. Additionally, only the primary, institution-level account for each platform was included. Smaller accounts with a specific topic or departmental focus (e.g. surgery) were excluded to ensure consistency across institutions and to focus on content disseminated through hospitals’ largest and most broadly followed SoMe channels. We abstracted account followers as of spring 2024.

The primary study author, an injury prevention expert (MA), trained the study coordinators (JG, ZF) on mechanisms of injury and reviewed multiple SoMe posts with each coordinator to calibrate classification decisions and ensure consistent classification of posts. These three authors then trained the team of data abstractors through structured meetings and discussion of coding decisions, and maintained ongoing availability to address questions throughout the abstraction process. Abstractors systematically reviewed all posts during the study period from all eligible accounts. Each post was reviewed by a single data abstractor. Data were recorded using a standardized electronic abstraction form and included hospital, SoMe platform, and date of post.

Each post was classified dichotomously as IP-focused or non-IP-focused. IP-focused posts were further subcategorized by injury mechanism, while non-IP-focused posts were subcategorized according to primary thematic content (e.g. cardiac, fundraising). When a post could reasonably be assigned to multiple subcategories, abstractors were instructed to select the single most appropriate category in their estimation.

Frequencies and descriptive statistics were used to characterize the hospitals and SoMe followers. Chi-square analyses were used to determine differences between groups. All data were analyzed with IBM SPSS Statistics^®^ (version 29.0.0.0 (241)). This study was declared exempt by the Institutional Review Board.

## Results

A total of 93 hospitals met initial inclusion criteria; however 11 were excluded because their SoMe accounts were not exclusively pediatric-focused, leaving 82 hospitals for analysis (Table 1). All included hospitals maintained a FB account (*N* = 82, 100%), 73 (89.0%) had an X account and 71 (86.6%) had an IG account as of January 1, 2023. Most hospitals maintained accounts on all three platforms (*N* = 69, 84.1%). Median follower counts were highest on FB (53,500; IQR 25,750 − 124,250), followed by IG (12,415; IQR 6,462 − 22,172), and X (10,800; IQR 5,840 − 24,192).

The majority of hospitals were verified level I pediatric trauma centers (*N* = 78, 95.1%), while 50 hospitals (61.0%) hosted a pediatric surgery fellowship, 40 (48.8%) were affiliated with IFCK, and 18 (22.0%) were associated with a past or current CDC ICRC. Only 10 hospitals (12.2%) had all 4 IP-related affiliations.

**Table 1:** Hospital SoMe Accounts & Affiliations

Type of SoMe Account	*N*	%
Facebook	82	100.0%
X	73	89.0%
Instagram	71	86.6%
All 3 accounts	69	84.1%
**Number of Followers**	**Median**	**IQR**
Facebook	53,500	25,750 − 124,250
Instagram	12,415	6,462 − 22,172
X	10,800	5,840 − 24,192
**IP Affiliations**	**N**	**%**
Level I pediatric trauma center	78	95.1%
Pediatric surgery fellowship	50	61.0%
IFCK	40	48.8%
CDC ICRC	18	22.0%
1 IP affiliation	25	30.5%
2 IP affiliations	20	24.4%
3 IP affiliations	27	32.9%
4 IP affiliations	10	12.2%

SoMe, social media; IQR, interquartile range; IP, injury prevention; IFCK, Injury Free Coalition for Kids; CDC ICRC, Centers for Disease Control and Prevention Injury Control and Research Center

A total of 55,339 posts were analyzed. The largest proportion of posts were on FB (*N* = 22,900, 41.4%), followed by X (*N* = 16,897, 30.5%) and IG (*N* = 15,542, 28.1%).

Of all posts, 3,863 (7.0%) were pediatric IP-focused (Table 2). The most frequently represented IP topics included mental health/suicide prevention, miscellaneous injury-related topics not captured by pre-defined categories (e.g. dog bites, child abuse, toy safety), poisonings, and child passenger safety. The majority of posts (*N* = 51,472, 93.0%) were not IP-focused. Nearly half of all posts were related to general publicity and goodwill (*N* = 23,533, 42.5%). Among disease-specific topics, the most frequently represented categories were cardiac conditions, cancer, and neonatal diseases.

**Table 2:** Content of 55,339 Social Media Posts

Topic	*N* (%)	% of All Posts
**Injury Prevention**	3,863	7.0%
Mental health/suicide	479 (12.40%)	0.87%
Other^a^	445 (11.52%)	0.80%
Poisonings^b^	436 (11.29%)	0.79%
Child passenger safety	358 (9.27%)	0.65%
Safe sleep	281 (7.27%)	0.51%
Water safety/drowning	267 (6.91%)	0.48%
Other transportation safety^c^	254 (6.58%)	0.46%
Multiple injury prevention topics	249 (6.45%)	0.45%
General injury prevention awareness	223 (5.77%)	0.40%
Sports	205 (5.31%)	0.37%
Burns	178 (4.61%)	0.32%
Firearms	147 (3.81%)	0.27%
Fireworks	104 (2.69%)	0.19%
Helmets	91 (2.36%)	0.16%
Concussions	55 (1.42%)	0.10%
Choking	45 (1.16%)	0.08%
Falls	33 (0.85%)	0.06%
Furniture tip overs	13 (0.34%)	0.02%
**Non-Injury Prevention**	51,472	93.0%
General publicity/goodwill	23,533 (45.72%)	42.53%
Other-medical	10,319 (20.05%)	18.65%
Fundraising	5,025 (9.76%)	9.08%
Other-non-medical	4,646 (9.03%)	8.40%
Cardiac	2,101 (4.08%)	3.80%
Cancer	1,848 (3.59%)	3.34%
Neonatal	1,803 (3.50%)	3.26%
Infectious disease/immunizations	1,493 (2.90%)	2.70%
Orthopedics	704 (1.37%)	1.27%

^a^Includes topics such child abuse, dog bites, toy safety, etc

^b^Includes button batteries, laundry detergent pods, opioids, medications, edibles, etc

^c^Includes teen driving, vehicular heatstroke, pedestrian, ATVs, etc

Posting frequency varied widely across hospitals, ranging from 67 to 2,954 posts per year (median 582; IQR 356.75-902.75). The proportion of posts focused on pediatric IP also varied substantially, with some hospitals having no IP-related posts and others dedicating up to 19.7% of their SoMe to pediatric IP content. However, in some instances, hospitals with higher overall posting volume had greater absolute numbers of IP-related posts even when those represented a smaller proportion of total content (median 31.5, IQR 13.75-57.00).

All hospitals with multiple eligible SoMe accounts had cross-posting across platforms. However, the proportion of posts focused on IP-content differed significantly by platform. Of the respective platforms, IP content was most frequently observed on X (*N* = 1,372, 8.1%) compared to FB (*N* = 1,671, 7.3%) and IG (*N* = 820, 5.3%) (***X***^2^ = 106.845, p-value < 0.001).

Hospitals with a greater number of IP-oriented affiliations posted significantly more pediatric IP content (***X***^2^ = 119.800, p-value < 0.001). Individual affiliations with a pediatric surgery fellowship and IFCK were also associated with higher numbers of IP-related SoMe content (Table 3). There was no significant association observed for affiliation with a level I pediatric trauma center or CDC ICRC.

**Table 3:** Hospital Affiliations with IP-Oriented Organizations and IP-Content on SoMe

Organization	X^2^	*p*-value
Pediatric surgery fellowship	93.793	< 0.001
IFCK	4.563	0.033
Level I pediatric trauma center	1.146	0.284
CDC ICRC	0.48	0.827

SoMe, social media; IFCK, Injury Free Coalition for Kids; CDC ICRC, Centers for Disease Control and Prevention Injury Control and Research Center

## Discussion

This cross-sectional analysis of SoMe content from leading US children’s hospitals found that pediatric IP messaging comprised a small proportion of overall content, representing only 7.0% of all posts and supporting our primary hypothesis. Despite widespread use of SoMe by both healthcare institutions and the general public including parents and caregivers of children seeking health-related information, the relative scarcity of IP-focused content suggests that hospitals are not fully leveraging SoMe platforms to address the leading cause of morbidity and mortality in children [1, 3]. This gap represents a substantial missed opportunity for public health education and advocacy.

We also observed significant differences in IP messaging across SoMe platforms. Although hospitals cross-posted content, IP posts were most common on X and least common on IG. This may reflect differences in platform audiences and goals. While FB and IG are often used to engage patients, families, and communities, X historically served for professional networking, dissemination of research, and policy engagement. As a result, hospitals may view IP content as more aligned with the audience and objectives of X than with other platforms.

Our findings highlight a clear mismatch between the burden of pediatric injuries and the attention it receives on children’s hospitals’ SoMe platforms. Preventable injuries remain the leading cause of death and disability among children in the US [11–12]; yet the majority of SoMe content focused on publicity, goodwill, and non-injury-related health conditions. This disparity persisted even when restricting analysis to medically focused content. For example, although cancer is the third leading cause of pediatric deaths after firearms and motor vehicle crashes respectively [12], SoMe posts related to cancer (*N* = 1,848, 3.34% of all posts) outnumbered those addressing firearms IP (*N* = 147, 0.27% of all posts) by more than ten-fold and exceeded posts related to combined child passenger safety and other transportation safety (*N* = 612, 1.11% of all posts) by approximately three-fold. Notably, all predefined medical categories (cardiac, cancer, neonatal, infectious diseases, and orthopedics conditions) were more frequently represented than any individual IP topic. This pattern mirrors previously described disparities in pediatric research funding, in which conditions with lower mortality burden often receive disproportionately greater attention [13].

Even within IP-related content, the mechanisms responsible for the most fatalities were not consistently prioritized. For instance, sports injuries (*N* = 205, 0.37% of all posts) and burns (*N* = 178, 0.32% of all posts) were addressed more frequently than firearm-related IP, despite the latter representing the leading cause of death among children. These findings suggest that not only is IP underrepresented overall, but the distribution of IP messaging may not align with epidemiologic risk.

Hospitals with stronger ties to IP-oriented organizations, particularly those with pediatric surgery fellowships and affiliation with IFCK, were more likely to post IP-related content. These associations may reflect differences in institutional prioritization of IP. Pediatric surgery fellowships are associated with substantial academic and research infrastructure, which may increase awareness of injury epidemiology and support dissemination of prevention messaging. Similarly, affiliation with IFCK reflects a demonstrated commitment to pediatric injury prevention through community engagement, education, and advocacy. IFCK has also increasingly emphasized SoMe as a tool for injury prevention outreach through member training and coordinated large SoMe campaigns such as National Injury Prevention Day. In contrast, affiliation with a Level I pediatric trauma center or CDC ICRC was not associated with increased pediatric IP messaging. Collectively, these findings suggest that institutional priorities, leadership engagement, and organizational commitment to pediatric IP may influence hospital SoMe communication strategies. Additionally, IFCK maintains an exclusive focus on pediatric injury prevention, whereas CDC ICRCs often encompass a broader injury prevention portfolio that includes both pediatric and adult populations. Differences in organizational scope may contribute to the variation in pediatric IP-related social media content observed across affiliations.

We also observed substantial variability across hospitals in both overall frequency of SoMe activity and the proportion of IP-related content. Some hospitals infrequently posted or did not post any IP-related content, whereas others dedicated nearly one-fifth of posts to IP. This heterogeneity likely represents the diversity of children’s hospitals’ approach to SoMe, institutional priorities, and dedicated SoMe resources. In many institutions, SoMe accounts are managed by communications or marketing teams with varying levels of clinical input. Additionally, if hospitals primarily view SoMe as a tool for brand promotion, positive community engagement, and fundraising, institutions may preferentially highlight positive and noncontroversial topics while avoiding sensitive issues such as firearm injury, suicide, and cancer. However, such an approach may limit the potential public health impact of these platforms.

Taken together, these findings underscore the potential of SoMe as an underutilized tool for pediatric IP. Given its broad reach, low cost, and increasing use among caregivers seeking health information, SoMe represents a promising platform for dissemination of timely, actionable safety messaging [4, 6]. Prior studies have shown that digital health communication can influence knowledge and health behaviors when content is effectively designed and targeted [5]. Greater integration of evidence-based IP messaging into children’s hospitals SoMe strategies could enhance public awareness and potentially reduce injury risk.

This study has several limitations. First, some SoMe content may have been unavailable for analysis as some organizations, including hospitals, have completely deleted their accounts or gone inactive on X following changes in 2023 [14]. Additionally, individual posts, including those addressing potentially controversial topics such as secure firearm storage, safe sleep, and vaccines, may have been removed after publication. However these lost opportunities should not have tangibly impacted the vast overshadowing of IP SoMe content by other topics, our primary focus of this study. Additionally, hospitals may maintain multiple SoMe accounts on the same platform, some of which may focus more heavily on IP. We intentionally limited our analysis to primary institution-level accounts because they typically have the largest audiences and greatest potential reach. However, this approach may have underestimated the overall volume of IP-related content generated by children’s hospitals if IP messaging was concentrated within smaller departmental or program-specific accounts. Additionally, other SoMe accounts such as TikTok and YouTube were not analyzed. Further, content classification relied on manual data abstraction and may be subject to misclassification bias. For example, some posts could reasonably fit multiple categories, such as a post addressing secure firearm storage as a strategy to reduce suicide risk. In addition, each post was reviewed by a single trained abstractor, and inter-rater reliability was not formally assessed. Although standardized training and ongoing investigator support were used to promote consistency, variability in classification decisions may have occurred. However the primary dichotomization of IP vs. non-IP content was straightforward, and standardized procedures were used to enhance consistency. Additionally, this study evaluated the presence of IP messaging but did not assess audience engagement or effectiveness of posts in influencing knowledge, attitudes, or behavior. Finally, these findings reflect SoMe activity during 2023, and may not capture evolving trends in the intervening years.

## Conclusion

In this national analysis of children’s hospitals’ SoMe content, pediatric IP messaging comprised a small fraction of overall posts, despite preventable injuries being the leading cause of death in children. These findings highlight a critical opportunity to better align children’s hospital SoMe strategies with public health priorities. Given the reach of SoMe, these organizations are well-positioned to expand IP messaging to caregivers and communities. Integrating evidence-based IP content into routine SoMe communications and leveraging periods of increased injury risk may further enhance the impact of these efforts. Future research should investigate strategies to increase the quantity and quality of IP content from children’s hospitals, identifying the most effective strategies for delivering IP messaging, and evaluating the impact of these efforts on caregiver knowledge and behavior, and child health outcomes.

## Data Availability

Data is available upon email request to the corresponding author.

## Declarations

### About this supplement

This article has been published as part of Injury Epidemiology Volume 13 Supplement 1, 2026: Proceedings of the 30th Annual Injury Free Coalition for Kids^®^ Conference: Forging New Frontiers: injury Prevention in Evolving Environments. The full contents of the supplement are available online at https://link.springer.com/journal/40621/volumes-and-issues/13-1/supplement.

## Abbreviations

CDC: Centers of Disease Control and Prevention
FB: Facebook
ICRC: Injury Control and Research Center
IFCK: Injury Free Coalition for Kids
IG: Instagram
IP: Injury prevention
SoMe: Social media
X: Twitter/X
